# Investigating the Application of Liposomes as Drug Delivery Systems for the Diagnosis and Treatment of Cancer

**DOI:** 10.1155/2021/3041969

**Published:** 2021-09-01

**Authors:** Latifa W. Allahou, Seyed Yazdan Madani, Alexander Seifalian

**Affiliations:** ^1^School of Pharmacy, University of Nottingham, Nottingham NG7 2RD, UK; ^2^School of Pharmacy, University of Nottingham Malaysia, Semenyih, Selangor, Malaysia; ^3^Nanotechnology and Regenerative Medicine Commercialisation Centre (NanoRegMed Ltd.) London BioScience Innovation Centre, 2 Royal College Street, London NW1 0NH, UK

## Abstract

Chemotherapy is the routine treatment for cancer despite the poor efficacy and associated off-target toxicity. Furthermore, therapeutic doses of chemotherapeutic agents are limited due to their lack of tissue specificity. Various developments in nanotechnology have been applied to medicine with the aim of enhancing the drug delivery of chemotherapeutic agents. One of the successful developments includes nanoparticles which are particles that range between 1 and 100 nm that may be utilized as drug delivery systems for the treatment and diagnosis of cancer as they overcome the issues associated with chemotherapy; they are highly efficacious and cause fewer side effects on healthy tissues. Other nanotechnological developments include organic nanocarriers such as liposomes which are a type of nanoparticle, although they can deviate from the standard size range of nanoparticles as they may be several hundred nanometres in size. Liposomes are small artificial spherical vesicles ranging between 30 nm and several micrometres and contain one or more concentric lipid bilayers encapsulating an aqueous core that can entrap both hydrophilic and hydrophobic drugs. Liposomes are biocompatible and low in toxicity and can be utilized to encapsulate and facilitate the intracellular delivery of chemotherapeutic agents as they are biodegradable and have reduced systemic toxicity compared with free drugs. Liposomes may be modified with PEG chains to prolong blood circulation and enable passive targeting. Grafting of targeting ligands on liposomes enables active targeting of anticancer drugs to tumour sites. In this review, we shall explore the properties of liposomes as drug delivery systems for the treatment and diagnosis of cancer. Moreover, we shall discuss the various synthesis and functionalization techniques associated with liposomes including their drug delivery, current clinical applications, and toxicology.

## 1. Introduction

Cancer, the disease elicited by the uncontrolled division of cells within the body, was responsible for approximately 8 million deaths worldwide in 2007, accounting for 13% of total deaths [1]. In 2018, 9.6 million deaths worldwide were estimated to be due to cancer [2]. Furthermore, the deaths caused by cancer are projected to increase with 12 million deaths estimated to occur in 2030. As a result, the development of effective cancer monitoring, diagnostics, and treatment is vital, yet remains a challenge. The current, available treatments for cancer include, but are not limited to, radiotherapy, surgery, and chemotherapy [3]. Despite the associated limitations and poor efficacy, chemotherapy remains the most common treatment for cancer [4]. The clinical employment of conventional chemotherapeutic agents has been restricted by their reduced efficacy. Cytotoxic cancer drugs possess the ability to act nonspecifically on both healthy and cancerous tissues in clinical use resulting in limited therapeutic drug dosages due to their toxic side effects on healthy organs [5, 6]. Therefore, drug doses cannot be sufficiently altered due to their lack of tissue specificity which hinders treatment.

One of the barriers to the treatment of cancer using conventional chemotherapeutics is the mutated characteristics of the target cancer cells which render them inaccessible. Furthermore, chemotherapeutic agents may lack the adequate stability and solubility characteristics necessary for efficacy at the site of action [7, 8]. Solid tumours have both physiological and biological factors that demand the formulation of an effective drug delivery system. These challenges include the mononuclear-phagocyte system (MPS) and the surrounding hypoxic environment [9]. Therefore, it is imperative that improvements are made to the current delivery of anticancer drugs to combat their toxicity and amplify half-life and selectivity for target tissues whilst diminishing serious side effects and the duration of treatment [10, 11]. Developments in the field of nanotechnology have been applied to medicine with the aim of overcoming the aforementioned obstacles in drug delivery. Nanoparticles serve as a paradigm for these developments as they offer solutions to the challenges associated with anticancer agents [12, 13].

Nanoparticles are particles ranging between 1 and 100 nm in size; they may be conjugated with drugs and utilized for drug delivery to enhance drug treatment [14]. The use of nanoparticles is advantageous for the diagnosis and treatment of cancer as they are long acting and have highly efficacious bioactivity and greater penetration within cells. In Addition, nanoparticles have modifiable release rates and cause fewer side effects to healthy organs [11]. Other nanotechnological developments include nanocarriers. The four main categories of nanocarriers are micelles, dendrimers, protein-based nanocarriers, and liposomes. They are capable of entrapping drugs within their matrix. Although they can be considered as nanoparticles, organic nanocarriers such as liposomes may deviate from the standard size range that is associated with nanoparticles as they can be larger than 100 nm [15].

**Figure 1:**
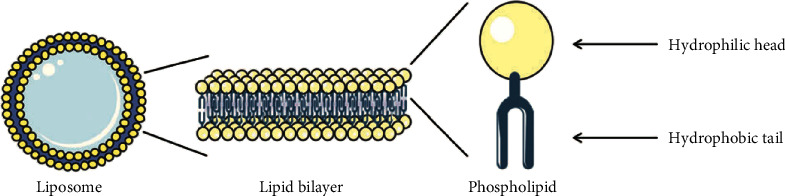
Diagram of a liposome including the amphiphilic phospholipid component that forms its concentric lipid bilayers [7].

In 1964, Dr. Alec D Bangham discovered liposomes at the Babraham Institute at the University of Cambridge [16]. The name liposome originates from the union of two Greek words: “Lipos” meaning fat and “Soma” meaning body [17]. Liposomes are small spherically shaped artificial vesicles containing one or more concentric lipid bilayers that encapsulate an aqueous core with particle sizes that range between 30 nanometres and several micrometres (Figure 1). There is a plethora of factors such as magnitude, lipid constitution, preparation method, and surface charge that influence the properties of liposomes. For instance, the choice of bilayer constituents bears consequences on the charge and rigidity of the bilayer; unsaturated phospholipids such as soybean phosphatidylcholine yield bilayers with higher permeability, whereas saturated phospholipids such as dipalmitoylphosphatidylcholine yield impermeable rigid bilayers. The lipid bilayer may be comprised of natural or synthetic phospholipids. Liposomes that are composed of natural phospholipids are considered to be poorly immunogenic, biologically inactive, and have low innate toxicity [18, 19].

Lamellarity, size and preparation method are the ways in which liposomes can be classified. Unilamellar vesicles have diameters ranging between 50 and 250 nm; they are characterised by one lipid bilayer that encapsulates an aqueous core. Inversely, multilamellar vesicles have diameters ranging between 1 and 5 micrometres, and they are distinguished by their multiple concentric “onion-skin” bilayers [20]. Liposomes are formed when amphiphilic molecules spontaneously form physically stable sealed structures in the presence of water. Upon hydration in an aqueous solution, these phospholipids that possess polar head groups face inwards towards the aqueous core within the cell and outwards towards the surrounding aqueous environment. Conversely, the hydrophobic fatty acid chains face inwards as they are repelled by water, thus creating a hydrophobic environment. Liposomes have a diameter that varies between 1 nm and several microns. For liposomal formulations to be considered as nanostructure systems and clinically utilized, they must be within the submicron ultra-filterable range which is less than 200 nm in size [16, 21]. Numerous methods of liposome synthesis have been documented, these methods may be segregated into two different categories: passive and active loading techniques. Passive loading techniques can be further subdivided into solvent dispersion methods, mechanical dispersion methods, and detergent removal methods. Active loading techniques utilize both a difference in potential across the membrane of liposomes and a pH gradient to load preformed liposomes with drug molecules [19, 22].

**Figure 2:**
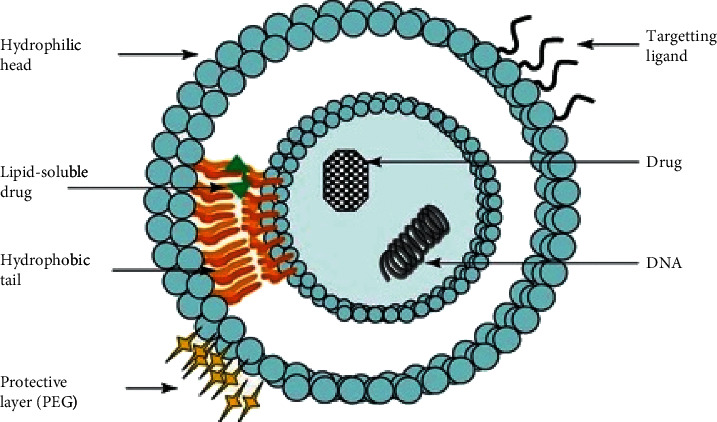
Diagram illustrating a liposome entrapping both hydrophilic and hydrophobic drugs in the aqueous core and lipid bilayer, respectively. The surface of the liposome allows for the addition of targeting ligands and a polyethylene glycol coating for active and passive targeting, respectively [8].

Liposomes provide a higher drug payload per particle by encapsulating a diverse range of therapeutic and diagnostic agents and they offer protection to the drugs they encase from metabolism [23]. The varying lipophilicities of different drugs determine where they can be encapsulated within the liposome. Hydrophilic drugs with log *P* value < −0.3 are entrapped exclusively within the aqueous core, whilst strongly lipophilic drugs with log *P* value > 5 are encapsulated almost entirely within the lipid bilayer (Figure 2). Drugs that are capable of partitioning between the lipid bilayer and the aqueous core possess an intermediate log *P* value (1.7 < log  *P* < 4) [20]. Liposomes are being employed to facilitate intracellular delivery of anticancer agents within the pharmaceutical industry as they are able to elongate the retention time of the entrapped drug within cancer cells. The issues concerning the off-target side effects may be resolved by liposomes as they enhance the pharmacological and pharmacokinetic profile of anticancer drugs [24]. Moreover, liposomal formulations may improve in vivo activity of anticancer agents. The anticancer drug cytosine arabinoside was delivered to mice with L1210 leukaemia within a liposomal formulation, the resulting in vivo activity was improved, and the survival times of the mice were significantly increased [25]. For these reasons, liposomes are favoured for their advantageous properties as drug delivery systems for the in vitro and in vivo delivery of biologically active substances [26].

Owing to their distinctive properties, such as low toxicity, biocompatibility, and biodegradability, liposomes have been recognized as attractive potential carriers for therapeutic agents throughout the past fifty years. Liposomes enable sustained drug release and produce less systemic toxicity in comparison with free drug [27]. Furthermore, due to their amphiphilic phospholipid bilayers, liposomes share a resemblance to biological membranes [28]. Liposomes are therefore considered to be ideal carrier systems and have been recognized as the most successful drug carrier system thus far [29]. In addition, the barrier liposomes form protects their contents from the oxidation caused by free radicals and degradation caused by bile salts, gastric acid, and alkaline solutions located within the mouth and stomach. Therefore, the phospholipid bilayer shields the liposomal contents throughout their delivery to the intended target organ or system where the contents may be released [30].

Liposomes may be manufactured on a larger scale with a magnitude ranging between 50 nm and 150 nm favouring the enhanced permeability and retention effect (EPR), which enables the liposomes to extravasate and accumulate into tumour tissues from blood vessels. Although conventional liposomes are advantageous as carrier systems, they are subjected to various obstacles throughout delivery. One of the challenges faced by conventional liposomes is recognition by the host's immune systems, namely, the MPS. Therefore, they may face rapid clearance by the MPS and scavenging by the spleen and liver. The action of these clearance mechanisms affects the optimization of the pharmacokinetic profile of the drug encapsulated within the liposome [7]. Therefore, liposomes must be equipped with the appropriate functionalities such as enhanced accumulation and cellular internalisation at target tissues, longer systemic circulation, and drug delivery that is target-specific to the organelle; this may be accomplished by modifying the surface of the liposome [31]. Grafting with various targeting ligands such as peptides, antibody fragments, and aptamers has aided in the efficient targeting of liposomes, their endocytosis, and therapeutic response [32].

Consequently, the engineering of liposomes with functional moieties enables them to enhance their target-specific delivery, prolong circulation time, enhance intracellular penetration, and provide contrast enhancement for image-guided therapy. One example of the most widely utilized polymers is polyethylene glycol (PEG); liposomes functionalized with this hydrophilic polymer are referred to as “stealth liposomes” as they sterically stabilize the liposome, decreasing particle aggregation and recognition by opsonins [7]. Various biomedical applications of liposomes have already been approved for clinical use such as the liposomal formulation of the anticancer drug doxorubicin modified with the addition of PEG(Doxil®), the first liposome approved by the Food and Drug Administration FDA, whilst other liposomes are in still clinical trials [16, 33].

This review summarizes the properties liposomes possess and their method of synthesis. Furthermore, the functionalization techniques, toxicology, and drug delivery of liposomes have been outlined and their clinical application has been explored for cancer treatment and diagnosis.

## 2. Aims and Objectives

The aim of this systematic review is to investigate the various available applications of liposomes as nanoscale drug delivery systems for the treatment and diagnosis of cancer. We shall explore the benefits and acknowledge the limitations associated with the utilization of liposomes. The diverse approaches employed for their synthesis and functionalization shall be considered including toxicology studies.

## 3. Methods

To achieve an effective data collection process, the research implemented a repetitious procedure that employed inclusion and exclusion criteria as well as a search strategy and study selection.

### 3.1. Research Approach

Articles published up till January 25, 2021, were searched utilizing the universal bibliographic electronic databases PubMed and Science Direct. The keywords comprised “liposome,” “synthesis,” “functionalization,” “nanoparticle,” “cancer,” “chemotherapy,” “anticancer,” “stealth,” “surface,” “PEGylation,” “targeting,” and “nanomedicine.”

### 3.2. Study Selection

We have chosen to evaluate the eligibility of the articles through primary screening and secondary screening. The primary screening involved examining the abstract followed by the main text and the secondary screening involved the examination of the significance of the main text and results.

### 3.3. Inclusion Criteria

The following features were met by the eligible studies that were incorporated in the current review: articles detected using the aforementioned keyword search, articles containing an adequate amount of information regarding the diverse synthesis and functionalization techniques of liposomes, articles written in the English language, articles that explore the properties of liposomes as drug delivery systems for the diagnosis and treatment of cancer, and articles that discussed the biomedical application of liposomes. Furthermore, it should be noted that review papers were included within this systematic review.

### 3.4. Exclusion Criteria

The following features were met by the ineligible studies that were eliminated in the current review: articles not written in the English language, articles that that did not explore the properties of liposomes as drug delivery systems for the diagnosis and treatment of cancer, and articles that discussed the agricultural application of liposomes.

### 3.5. Data Collection

The following data comprising the characteristics, synthesis, functionalization, clinical applications, toxicity, and potential of liposomes as drug delivery vehicles for the treatment and diagnosis of cancer were extracted. Moreover, we have conducted and inspected the data extraction to ensure all data collected were appropriate and relevant.

## 4. Methods for Preparation of Liposomes

Liposomes may be synthesized utilizing a diverse range of methods. However, the standard procedure for their preparation generally involves the production of a thin film formed from the vaporization of organic solvents containing phospholipids. The thin film layer becomes hydrated, dispersing the phospholipids. Bilayer sheets form with the inclusion of hydrophobic compounds due to the incorporation of aqueous ingredients and an aqueous phase, which is followed by the involvement of adequate amounts of thermal energy and shaking which promote mechanical agitation and sonication or a combination of these techniques. Liposomes are formed once the bilayer sheets are extracted from the bulk materials [34].

Active and passing loading encapsulation may be employed to form liposomes. Passive loading encapsulation involves the entrapment of compounds during vesicle formation, whilst active loading encapsulation includes the insertion of bioactive compounds into intact vesicles utilizing a driving force potential attained by calcium acetate and ammonium sulphate, for instance. However, the aforementioned preparation methods employ the use of toxic solvents including ethanol, ether solution, methanol, chloroform, and detergents to enhance the solubility of hydrophilic and hydrophobic ingredients [35, 36]. For the purpose of this review, passive loading techniques shall be discussed, these are subdivided into mechanical dispersion methods, solvent dispersion methods, and detergent removal methods.

### 4.1. Mechanical Dispersion Methods

Mechanical dispersion methods include lipid-film hydration via hand and nonhand shaking or freeze-drying, sonication, French pressure cell, membrane extrusion, freeze-thawed liposome, microemulsification, and dried reconstituted vesicles.

#### 4.1.1. Lipid-Film Hydration via Hand and Nonhand Shaking or Freeze-Drying

**Figure 3:**
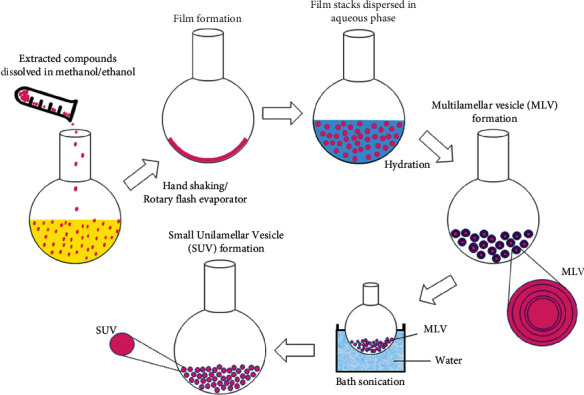
An illustration of the preparation process for synthesizing liposomes in a stepwise manner [34].

Lipid-film hydration is the most common mechanical dispersion method, involving the production of a thin film consisting of a phospholipid and cholesterol membrane which is derived from the evaporation of the organic solvent of the solution containing phospholipids and cholesterol. The evaporated solution is hydrated with a phosphate buffer solution; this process along with vertexing and occasionally, sonication, yields liposomes. Furthermore, to produce small unilamellar vesicles (SUV) from multilamellar vesicles (MLV), this process may be amalgamated with membrane extrusion (Figure 3). However, this method may leave traces of the organic solvent within the final product [22].

#### 4.1.2. Sonication

Sonication is a widely used simple method for the preparation of liposomes; it can be used to synthesize SUV from MUV [37]. This method involves placing MLV into a bath sonicator or disrupting them with the use of a probe sonicator. The application of ultrasonic irradiation reduces vesicle size by providing energy for the lipid suspension of MLV. The main drawback of this method is the poor encapsulation efficiency as a result of the low internal volume, potential degradation of the phospholipids including the compounds intended for encapsulation, large molecule eradication, and the additional presence of MLV [18, 26].

#### 4.1.3. French Pressure Cell

SUV may also be produced from MLV via the French pressure cell method which passes the desired liposomes through a small orifice. According to a report conducted by Hamilton et al., 90 percent of the final solution produced contained the desired SUV when the first suspension was passed through the orifice again. The liposomes produced by this method provide an advantage as they are larger than the SUV produced by sonication. Disadvantages of this method include small working volumes and difficulties in achieving a high temperature [18, 22].

#### 4.1.4. Membrane Extrusion

The membrane extrusion method can be used when a homogenous mixture of SUV is desired, due to the duplicability and improved size control. In this method, liposome suspensions are passed through polycarbonate membranes containing a specific pore size several times. However, the SUV produced may have a small volume available for carrying [38, 39].

### 4.2. Solvent Dispersion Methods

Solvent dispersion methods include ethanol injection, ether injection, double emulsion, and reverse-phase evaporation.

#### 4.2.1. Ethanol Injection

The ethanol injection method produces SUV in a simple manner via the rapid injection of an ethanol-containing lipid solution into a buffer. Although the liposomes may be extracted by the evaporation of excess ethanol and water, the ethanol forms azeotrope with the addition of water which is difficult to remove completely, and it is highly possible that several biologically active macromolecules may inactivate in the presence of even low levels of ethanol. Another challenge is that obtaining a homogenous mixture of similar-sized liposomes is difficult with this method in comparison to others [18,40].

#### 4.2.2. Ether Injection

In contrast to the ethanol injection method, the ether injection method requires that phospholipid and cholesterol are dissolved in an ether solution. Moreover, the solution is injected gradually into water [18]. Liposomes are obtained once the solution is heated above the boiling point of the ether. One of the advantages of this method is that any larger undesired vesicles remaining in the solution may be easily filtered out. Furthermore, this method excludes the need for liposomes to undergo a multilamellar phase and a homogenous mixture is obtained via simple means [22].

#### 4.2.3. Double Emulsion

The double emulsion method is analogous to ethanol injection, as it creates liposomes utilizing solvents. However, this method employs the use of two dissimilar solvents to achieve greater homogeneity within the liposome solution. Liposomes in this method are synthesized by the addition of phospholipids to ethanol. The resulting solution, once agitated, is added to a second solvent containing glycerine. This method is favoured over just ethanol injection as a greater homogenous vesicle mixture may be produced with this method. In addition, it fosters a novel preparation method for liposomes for industrial application, as it generates large quantities of liposomes from elevated concentrations of phospholipids [41].

### 4.3. Detergent Removal Method

Detergent removal methods include dialysis, dilution, and column chromatography. Solutions containing phospholipids and cholesterol form mixed vesicles of varying magnitudes and shapes once detergents are added. Detergent removal can increase the size of the mixed micelles; however, the lipids form liposomes at a critical ratio of detergent to lipid. Detergent removal is an attractive method as it allows for the efficient production of unilamellar liposomes. Conversely, the effective removal of the detergent from the functional liposome is vital as it can result in macromolecular degradation. One of the several ways detergents can be removed is via dialysis. Dialysis removes micelles and detergent monomers from mixed micelle solutions by exploiting its diffusion across the dialysis membrane [22, 42].

## 5. Functionalization of Liposomes

### 5.1. Introduction to Functionalization

Liposomal formulations overcome the shortcomings of biodistribution and targeting in drug delivery and serve to protect their encapsulated contents from degradation. However, upon intravenous administration, these liposomes are rapidly and prematurely cleared from the systemic blood circulation by the MPS. This clearance mechanism has been previously implemented for the delivery of liposome encapsulated agents intended to treat local infections within the MPS. Alternatively, if the target site is beyond the MPS, clearance of liposomes from the blood circulation by macrophages is unfavourable for conventional liposomes utilized in chemotherapy. Furthermore, reductions in the biodistribution of liposomes are one of the consequences of clearance [20, 25]. The macrophages of the MPS located within the liver and spleen behave as phagocytotic scavengers and are responsible for clearing nanocarriers larger than 30 nm. Other factors influencing clearance include the magnitude of endothelial fenestrae, although they vary greatly [43]. Opsonins (serum proteins) bind to the surface of liposomes as they are deemed as foreign materials. The MPS recognizes the surface bound opsonins and initiates phagocytosis [20]. Other proteins that assist the MPS in their identification and eradication of liposomes are fibronectin and immunoglobulins [33]. Therefore, it can be assumed that the liposomal surface properties bear a significant impact on their clearance rate by cells of the MPS [44]. A plethora of strategies have been researched throughout the years to tackle obstacles in stability and to produce long-circulating liposomes through surface functionalization of liposomes. Functionalization refers to the assembly of various inorganic and organic materials via noncovalent bonds and covalent bonds in nanoscale [45].

### 5.2. The Application of Long-Circulating Liposomes

Moderate improvements were observed in circulation half-lives when liposomal vesicle sizes were reduced during initial approaches. Initial research examined the distinctions between biological membranes with a carbohydrate coating the surface and unaltered phospholipid membranes. The first long-circulating liposomes that can produce an effect without the MPS blockade were developed with the attachment of the monosialoglyprotein (GM1) to liposomes containing cholesterol and egg phosphatidylcholine. Reduced liposomal uptake into the liver and prolonged circulation half-lives were achieved with the replacement of sphingomyelin with egg phosphatidylcholine. The enhanced surface hydrophilicity of the liposomes derived from the attached gangliosides was suspected to be the cause of this mechanism [25]. Liposomal formulations grafted with GM1 circulated in the blood for several hours, and it was discovered that a diameter ranging between 90 and 200 nm results in prolonged blood retention and tumour tissue accumulation. Sialic acid was found to play a vital role in its MPS-avoiding effect; however, its extensive utilization is costly, leading to further investigations of alternative potential derivatives [20].

Both GM1 and the hydrophilic polymer PEG have previously exhibited success as polymers utilized to prolong the duration of circulation of liposomes [16]. However, the optimal strategy discovered was first described by scientists in the 1990s. Their experiment revealed that PEGylation of the liposome surface prolonged circulation time considerably and enhanced their stability by avoiding macrophages. The term “stealth” liposome was associated with long-circulating liposomes such as those sterically stabilized with PEG due to their ability to circumvent the immune system by concealing liposomes from macrophages. The presence of a steric barrier reduces the adhesion of blood components that lead to uptake by macrophages. The resultant in blood-circulation time in vivo was significantly enhanced. This is due to the fact that PEG chains form a hydrophilic film comprised of tightly bound water molecules that protect the liposomal surface by repelling serum protein interactions [16, 25, 26].

The linear polyether diol, PEG, is recognized for its attractive properties that permit its diverse clinical application such as solubility in both aqueous and organic mediums, biocompatibility, low toxicity, and immunogenicity as well as antigenicity. PEG molecules are favoured over GM1 as their structure and molecular weight may be manipulated freely for specific causes. Furthermore, PEG is more cost-effective and simpler in terms of the conjugation of polymers with lipids to form PEGylated liposomes [20]. To further prove the credibility of PEGylated liposomes, their half-life was compared to that of non-PEGylated liposomes. After PEGylation, the half-life of liposomes improved from a few hours to 45 hours. For this reason, the clinically approved liposomal formulation of the anticancer drug doxorubicin has undergone PEGylation to enhance the accumulation of liposomal doxorubicin in tumour sites for treatment. Although PEGylation prolongs circulation, it may impede target cell uptake due to steric hinderance induced by PEG [25, 26]. PEG lipids are incorporated into liposomes at 4–10% of total lipid to confer prolonged circulation and they have an optimal weight ranging between 2000 and 5000 Da. Once polymer quantities are congruent with the above values, protein interactions may be successfully impeded [16].

The preclinical study results of doxorubicin encapsulated liposomes were promising; this was also reflected in human clinical trials. After one week of injection, doxorubicin remained encapsulated within the circulating liposomes. Drug metabolites located at tumour sites indicated that drug release from the liposomes had occurred. Furthermore, superior drug absorption rates were observed in the doxorubicin encapsulated liposome, as it was 4–10 times more superior than the free drug used to treat the control group. The doxorubicin encapsulated liposome had a 10-fold higher drug concentration in lesions compared to the free drug [16]. The doxorubicin encapsulated liposome offers reduced cardiotoxicity and increased efficacy over free doxorubicin; this may be ascribed to passive targeting and a lower concentration of free drug at healthy tissues [8]. In Addition, a boron neutron capture therapy (BNCT) study conducted by a group of researchers at Kyungpook National University examined the delivery of high concentrations of water-soluble nido-carborane anion-loaded PEGylated liposomes. PEGylated liposomes were utilized as boron carriers in efforts to reduce MPS uptake and enhance drug delivery to tumours. The boronated liposomes exhibited profound tumour tissue penetration and demonstrated localization within the tumour cell cytoplasm. Furthermore, after a single dose and 20 minutes of neutron irradiation, the BNCT study achieved nearly complete tumour growth suppression [46].

### 5.3. The Application of Ligand-Targeted Liposomes

**Figure 4:**
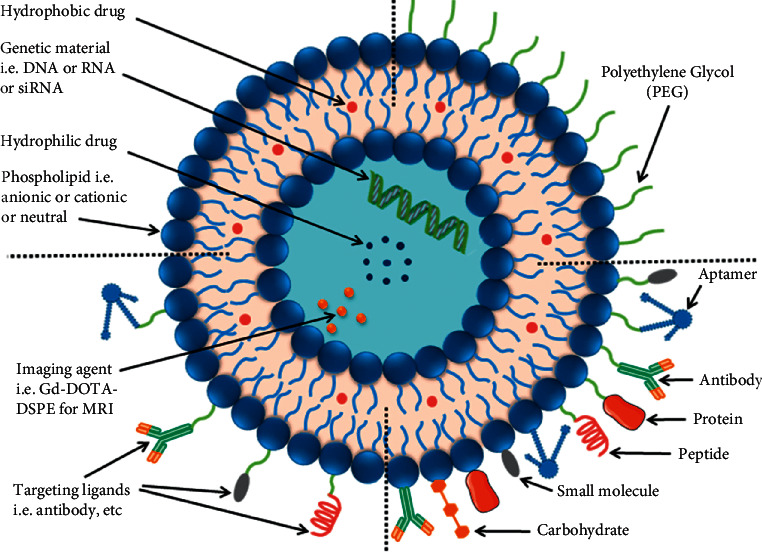
A diagram illustrating the potential for diverse functionalization of liposomes. Conventional liposomes lack surface modifications, or they can be coated with PEG on their surface. Various targeting ligands can be attached for active targeting and multiple targeting ligands can be added to the liposomal surface [24].

Liposomes with selectivity to diseased cells were desired during the early history of liposomes, especially if their interaction with cellular targets could initiate receptor-mediated endocytosis. Novel coupling strategies have been developed that involve the attachment of ligands to the terminus of PEG molecules on the surface of liposomes which resulted in enhanced in vivo mice lung tumour cell survival in comparison to nontargeted liposomal drugs [25]. Ligand-targeted liposomes for targeted drug delivery of anticancer agents at tumour sites are formed due to the functionalization of liposomal surfaces with suitable ligands that include, but are not limited to, peptides, aptamers, carbohydrates, antibodies, and their fragments and many more (Figure 4). These liposomes exploit and selectively recognize overexpressed receptors or antigens on the surface of target cancer cells and use them as docking sites. Their high selectivity for target cancer cells manifests as an increased accumulation of the encapsulated drug at tumour sites whilst disregarding healthy cells, resulting in a higher therapeutic index and drug efficacy [24, 26, 47–49]. Due to the fact that smaller amounts of the drug accumulate in healthy cells, drug toxicity and adverse side effects are subsequently reduced [50]. Various surface engineering techniques are utilized to functionalize liposomes with targeting ligands (Table 1). Ligands may be coupled via a spacer molecule to a hydrophobic anchor through cross-linking molecules. Hydrophobic anchors are necessary for ensuring stability during the insertion of conjugates into the liposomal bilayer. The hydrophobic anchor must have sufficient strength for binding a ligand such as an antibody and a spacer molecule such as PEG to the liposomal surface. Anchor choice relies on reactive group availability on the lipid, desired chemical bond type, and accessibility of heterofunctional cross-linking molecules. The general anchor of choice has been phosphatidylethanolamine due to the presence of its reactive amine and diverse acyl chain lengths with varying degrees of unsaturation [15].

Peptides are another example of targeting ligands that can be utilized for the targeted delivery of liposomes. A study conducted by researchers at Northeastern University indicated that the surface modification of PEGylated doxorubicin-loaded liposomes with octa-arginine (R8) peptide resulted in a significant increase in the delivery of doxorubicin compared to unaltered liposomes containing doxorubicin. Furthermore, the liposomes modified with R8 delivered more doxorubicin to the site of action which culminated in increased in vitro apoptosis of cancer cells, tumour growth suppression, and enhanced cytotoxicity in mice[55]. Similarly, aptamers are promising targeting ligands that can enhance the efficiency of PEGylated-liposomal doxorubicin (PLD). Researchers from Mashhad University of medical sciences demonstrated that the TSA14 aptamer PLD was able to target surface ligands of TUBO breast tumour cells. Aptamer modification resulted in the inhibition of tumour growth in mice and increased cytotoxicity and tumour levels of doxorubicin [56].

Lastly, another ligand coupling method includes “click” chemistry which involves the use of functional groups to functionalize liposomes to enhance drug delivery; polar azide groups were utilized in this study. Yunxin et al. successfully established PEGylated azide-functionalized liposomes containing drug nanocrystals as a novel targeted anticancer delivery system. The click reaction strain-promoted azide-alkyne cycloaddition (SPAAC) was chosen for the surface modification of the liposomes in this study. In order to attain targeted delivery of modified liposomes with SPAAC, complementary functional groups are incorporated into cancer cells to provide a target site for azide binding. This can be achieved with the glycan metabolic labelling technique which involves the incorporation of synthetic sugars with complementary “click” functional groups into cancer cells which eventually express these metabolised chemical groups in the form of glycoproteins on their cell surface. The metabolic rates of cancer cells exceed those of normal cells allowing for the abundant expression of these glycoproteins for binding with liposomes that possess the complementary azide groups [7].

## 6. Drug Delivery of Liposomes

**Figure 5:**
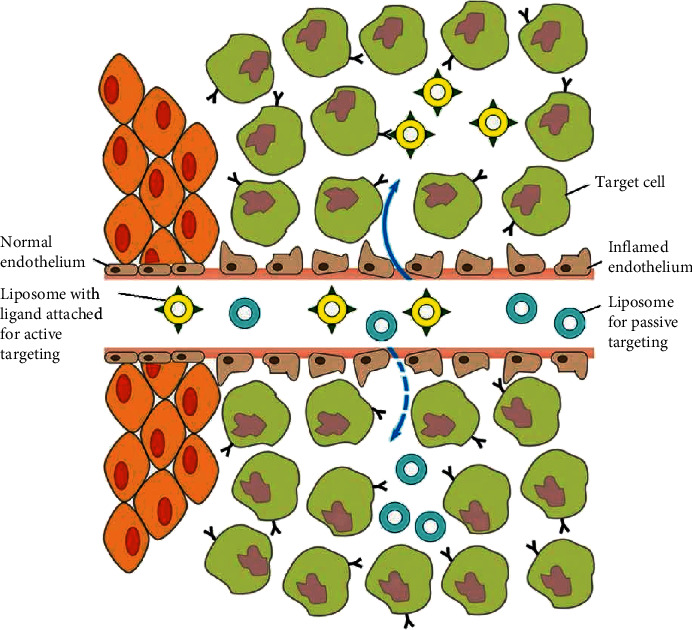
Diagram demonstrating the extravasation of liposomes into the interstitial fluid via cell gaps. Drug release occurs once ligands conjugated to liposomes bind to specific target cell receptors via active targeting. Local high-concentration drug release mediates passive targeting [8].

Numerous strategies have been developed for the purpose of producing liposomes that selectively target tumour cells and deliver anticancer agents to tumour sites. In this section, we will discuss both active and passive targeting techniques facilitated by the surface functionalization of liposomes (Figure 5) [24, 27].

### 6.1. Passive Targeting

Passive targeting is a strategy that depends solely on the pathophysiological characteristics of tumour tissues for drug targeting. Liposomal drug formulations translocate freely across the endothelium of capillaries into the interstitial fluid due to leaky tumour vessels. The pores that lie between the endothelial cells of tumour microvasculature vary considerably in size. Gap sizes between endothelial cells that line normal capillaries range between 5 and 10 nm, whereas the gap sizes between endothelial cells of tumour capillaries range between 100 and 780 nm which enables liposomes to engage in passive targeting. Consequently, ideal targeting can be obtained if liposomes are prepared with a size range appropriate for extravasation into tumour tissues and not normal tissues. The phenomenon that enables effective liposomal accumulation in tumours is termed as the EPR effect.

Due to the EPR effect, blood capillaries in cancerous tissues have greater permeability and a limited fluid return to the lymphatic circulation. The increase in vascular permeability may be attributed to the overexpression of regulatory angiogenic factors such as vascular endothelial growth factor (VEGF). Therefore, liposomes up to 400 nm in size and their encapsulated drugs can preferentially accumulate within the microenvironment of solid tumours owing to the inadequate lymphatic drainage of extravasated molecules [21, 27]. Furthermore, EPR is mutable as the diameter of vessel fenestrations varies. The consequent accumulation of liposomes in solid tumours provides improvements in drug delivery as there are higher local drug concentrations available [4]. The EPR effect may be optimized by preparing liposomes with particle sizes ranging between 40 and 200 nm as they have exhibited greater extravasation. Other improvements include the utilization of either external or internal stimuli to enhance the permeability of cancer cells [57]. Alternatively, the EPR effect may be enhanced with prolonged systemic circulation as it enables a greater number of blood passages through the target and extends interactions between liposomes and the target. As previously discussed, coating liposomes with PEG bears advantages such as avoidance of macrophagic uptake, however, grafting liposomes with PEG in these circumstances increases the longevity of liposomes within the blood resulting in improvements to the EPR effect [58, 59].

### 6.2. Active Targeting

In order to further minimize off-target side effects, different strategies have been employed to design actively targeted liposomes [27]. Active targeting involves directly targeting drug payloads at the target site [58]. Preparing actively targeted liposomes generally involves the conjugation of targeting ligands onto the surface of liposomes such as peptides, monoclonal antibodies, and aptamers [21, 60, 61]. Ligands may be attached to liposomes in a multitude of ways, including direct attachment to lipids or attachment at the terminal end of PEG chains. Ligand-lipid-PEG conjugated micelles may be incorporated into preformed liposomes via the postinsertion technique. Micelles and Stealth liposomes containing surface conjugated PEG form targeted liposomes upon incubation. One other commonly utilized approach is ligand incorporation into the step of liposome formulation [24]. These ligands can be utilized to exploit specific receptors and antigens expressed by cancer cells. Liposomes with targeting ligands attached can undergo endocytosis into cells by interacting with specific receptors on cell surfaces. Furthermore, in order to obtain an increased affinity for cell surface receptors, targeting ligands must be attached in adequate amounts. Examples of receptors that are overexpressed on numerous cancer cells include folate and transferrin receptors [21, 27]. Folic acid has been previously utilized as a targeting ligand as it can easily be conjugated onto nanocarriers; it possesses a high affinity for folate receptors which are overexpressed in a variety of cancers, including breast, lung, colon, and more. Low et al. researched the toxicity of doxorubicin-loaded PEGylated liposomes against FR + tumour cells, and they demonstrated that incubated cultures containing targeted doxorubicin-loaded PEGylated liposomes had a 45-fold greater uptake compared to untargeted liposomes [62].

Somatostatin receptor 2 is another attractive target for the selective delivery of liposomes as it is overexpressed in breast cancer cells. Bharti et al. produced diacerein (DN) loaded liposomes (DNL) conjugated with a synthetic analogue of somatostatin as the targeting ligand (SST-DNL). They demonstrated that SST-DNL had significantly higher antitumour efficacy in breast cancer models compared to DNL and free DN and increased apoptosis was observed in breast cancer cells in SST-DNL treated groups [63]. However, it should be acknowledged that both actively and passively targeted liposomes are distributed via the same passive distribution mechanism, denoting that increases in survival are primarily due to enhanced receptor-mediated liposomal uptake and not enhanced uptake of targeted liposomes by diseased cells. Furthermore, liposomes can be utilized as imaging probes as well as delivery vehicles for anticancer agents as they deliver anticancer agents such as doxorubicin and contrasting agents such as Magnevist® simultaneously within modified liposomes [64].

Drugs may be loaded actively or passively into liposomes. Active loading involves loading hydrophilic drugs following liposome formation. Passive loading refers to the direct encapsulation of lipophilic drugs into liposomes during vesicle formation [18]. One issue liposomes face includes the premature leakage of liposomal contents prior to uptake and binding at target sites. Therefore, drug release rates are vital aspects of drug delivery [25]. Due to the short-lived retention associated with liposomes after in vivo administration, several strategies have been implemented to resolve this issue. Cargo retention may be enhanced in liposomes possessing a solid-phase bilayer which is synthesized via the incorporation of sphingomyelin or cholesterol into liposomes. Another strategy aiming to control drug release rates suggests selecting drugs with physical characteristics favouring retention in liposomes. As previously mentioned, liposomes encapsulate both hydrophobic and hydrophilic drugs. Hydrophilic anticancer drugs entrapped within the aqueous cores of liposomes are released gradually over the span of several hours and days. However, the retention of strongly lipophilic drugs remains an issue as they are effortlessly released from liposomes. Studies have been conducted to enhance the retention of liposomal drugs such as the study conducted by Reshetov et al. that discovered that liposomes prepared with saturated phospholipids increased drug retention and blood circulation when compared with liposomes prepared with unsaturated phospholipids. Moreover, anticancer drugs with an intermediate log P are released rapidly from liposomes. This may be improved by forming molecular complexes in liposomes or by influencing the internal pH of liposomes, resulting in the exponential retention of drugs such as doxorubicin and daunorubicin (both weak bases). Conversely, liposomal retention may be achieved by converting drugs that are not weak bases to weak base prodrugs or by loading drugs at high intraliposomal concentrations beyond their solubility limits, increasing precipitation [26, 65].

Furthermore, there are limitations surrounding the transport of chemotherapeutics across cell membranes to intracellular targets, urging the need for modified liposomal delivery systems such as fusogenic liposomes that deliver drugs to the cytoplasm by exploiting a fusion mechanism utilized by the Sendai virus, independent of endocytosis [18, 25]. Another popular method of entry into the endosomal-lysosomal compartment is via the receptor-mediated endocytosis of targeted liposomes. In early experiments, receptor-mediated endocytosis and increased selective toxicity were demonstrated by targeted liposomal anticancer agents with antibodies attached (immune liposomes) [25]. Antigens can be incorporated into liposomes to form immune liposomes that behave as immunological adjuvants enhancing immune responses and antitumour activity. Despite the promising results obtained from in vitro studies using immune liposomes, they had poor in vivo applications [16].

Liposomal drug delivery systems are favoured over conventional drug delivery for topical and parenteral administration via local or systemic injections [26]. In order to improve the therapeutic outcomes of liposomes, triggered liposomal content release must occur upon the arrival of liposomes to target sites. There are two main trigger types: remote triggers include heat, light, and ultrasound and local triggers are inherent to the diseased site, including pH alterations and enzymes. Hyperthermia, a remote trigger, was used to deliver liposomal methotrexate which exhibited a fourfold increase in delivery to heated tumours compared with nonheated tumours. pH-sensitive liposomes formulated with lipid palmitoyl homocysteine can enhance drug release in local triggers such as primary tumour regions with a mildly acidic pH [25]. Stimuli-responsive liposomes offer an advantageous alternative for the selective release of drug payloads. The distinctive tumour microenvironment serves as an applied external stimulus or endogenous stimulus to trigger membrane destabilization derived from local defects for the controlled release of encapsulated liposomal drugs within a specific biological target. Various chemical activation approaches such as pH, redox, light, and enzyme have been employed to provide liposomes with stimuli-responsive features [7].

The encapsulation of drugs in liposomes changes their pharmacokinetics and biodistribution, greatly increasing treatment efficacy and reducing toxic effects. This is evidenced in preclinical and clinical studies using liposomal formulations of anticancer agents. Liposomes may also be applied in gene therapy by forming complexes with DNA, and for vaccinations as adjuvants that potentiate an immune response to vaccine antigens [16, 18].

## 7. Clinical Application of Liposomes

The early studies conducted by Morgan et al. were the primary demonstrations that liposomes were capable of accumulating in regions with increased vascular permeability via the EPR effect in humans. Morgan et al. demonstrated that indium 111-labelled liposomes could accumulate within solid tumours such as malignant lymphoma and Kaposi's sarcoma [25]. Since then, liposomal formulations constitute the largest cohort of clinically approved nanocarriers for anticancer drugs. The most widely recognized example is Doxil, which is PLD [15]. Doxorubicin encapsulated liposome was the first PEGylated-liposomal formulation to gain approval from the FDA in 1995 for the treatment of Kaposi's sarcoma in patients suffering from AIDS and recurrent ovarian cancer [26]. Doxorubicin encapsulated liposome exhibited enhanced drug retention as it forms a bilayer that is nonflexible at 37°C and below due to its optimal ratio of cholesterol and HSPC.

A remote loading approach that was developed first by Barenholz resulting in a high drug to lipid ratio using a transmembrane gradient that operates as a driving force for the efficient loading of amphiphilic weak base drugs such as doxorubicin. 15,000 molecules of doxorubicin per liposomal vesicle were systemically accumulated into the aqueous core of the liposome and more than 90% of the drug was present in a stable crystalline-like precipitate, liberated from osmotic effects. This loading approach allows for acceptable drug distribution rates in tissues, reduced drug efflux in the blood circulation, and increased retention. Gabizon et al. conducted a pilot clinical trial study with 15 cancer patients and compared doxorubicin encapsulated liposome to free doxorubicin. Clearance was greatly reduced in doxorubicin encapsulated liposome with 0.1 litres cleared per hour, compared to 45 litres cleared per hour with the free doxorubicin drug. Furthermore, doxorubicin encapsulated liposome displayed a reduced volume of distribution compared to the free doxorubicin drug. Moreover, there was 4–16 times greater concentration of doxorubicin within the tumours of patients who were administered doxorubicin encapsulated liposome.

Cardiotoxicity is one of the undesired side effects associated with doxorubicin treatment; this side effect was clinically reduced by doxorubicin encapsulated liposome as the entrapped doxorubicin cannot become bioavailable at the myocardium and cardiomyocytes [66]. At cumulative doxorubicin doses of 400–550 mg/m^2^, there is irreversible cardiotoxicity at an incidence of 7.5%; however, studies involving PLD demonstrated a reduced incidence of cardiac failure at doses even greater than 500 mg/m^2^. More evidence illustrating the ideal cardiac safety profile of PLD was discovered in prospective trials executed on patients with advanced gynaecological malignancies as the patients treated with PLD showed no myocardial damage which may enable treatment prolongation. Although, the PEGylation of liposomes prolongs blood circulation and alters the biodistribution of drugs in the body, significant incidences involving stomatitis have been reported in clinical trials evaluating the utilization of PLD and lipodox [26]. In the case of clinically approved lipid-based and liposomal products, parental administration routes are largely favoured, particularly intravenous administration routes. On the other hand, oral delivery routes are generally avoided due to the low bioavailability of encapsulated drugs that may arise as a result of GI degradation [25].

Targeted liposomes can increase the selective accumulation of liposomal drugs in target tissues. Targeting moieties that can be coupled to liposomes include carbohydrates, glycoproteins, and monoclonal antibodies (mAb) which create immunoliposomes. Anti-HER2 trastuzumab, Herceptin®, is the first humanized mAb that was granted approval by the EU and FDA for the treatment of metastatic breast cancer which is now widely used both in conjugation with chemotherapeutic agents and in isolation [20]. MCC465 is a doxorubicin-loaded targeted liposome conjugated to the F(ab')_2_ fragment of the human mAb, GAH for the treatment of stomach cancer cells and they share a resemblance with the pharmacokinetic profile of doxorubicin encapsulated liposome. They are still in early phase clinical trials and were capable of stabilizing disease in 10 out of the 18 patients treated [15]. Following the development of doxorubicin encapsulated liposomes, other liposomes have been approved and widely applied as nanocarriers for chemotherapeutic agents (Table 2) and some remain under investigation in clinical trials (Table 3) [26].

## 8. Toxicology Studies

Liposomes are recognized for their low intrinsic toxicity in comparison to other nanocarriers; this is mainly attributed to their natural phospholipid composition. Natural phospholipids are poorly immunogenic and biologically inactive [15, 20]. The phospholipids most commonly employed in the formation of liposomes include sphingomyelins and lecithin derived from soy and egg [109]. The surface charge and polymorphic state in an aqueous environment may be modified by different phospholipid head groups such as phosphatidylserine (PS) and phosphatidylcholine (PC). PS has an overall negative charge due to its phosphate group which is negatively charged and conjugated to a serine group.

Conversely, despite the notion that PC is a neutral molecule, it is considered zwitterionic due to the presence of a negatively charged phosphate group attached to a positively charged choline group [110]. Adams et al. conducted a study to examine the in vivo toxicity of five different liposomes synthesized with differing net surface charges. These liposomes were injected into the brains of mice and the results indicated that the liposomes containing phosphatidic acid (9 mol%) with a net negative charge were well tolerated in mice with minor haemorrhages and necrosis at the site of injection.

However, liposomes containing negatively charged dicetyl phosphate (9 mol%) had the most toxic effects including epileptic seizures and even rapid death. Alternatively, the liposomes with the least toxicity contained neutrally charged dipalmitoyl lecithin or lecithin (45 mol %), using these liposomes minimized morphological alterations and toxic reactions even after three repeated doses were administered to the mice. Anionic lipids were discovered to have a greater thrombogenic potential. Therefore, local tolerance studies and suitable platelet aggregation assays could be performed to prevent the induction of pharmacodynamic alterations or proaggregatory effects after the intravenous administration of liposomes at entry sites [110]. Therefore, the toxicity liposomes possess relates to their dose, surface properties, model type, and exposure time. Nevertheless, liposomes are considered to have low toxicity and are biologically inert [111].

## 9. Conclusion

Liposomes constitute the majority of clinically approved nanocarriers for anticancer agents. Liposomes have been esteemed for their favourable attributes; they provide a wealth of opportunities for their extensive therapeutic pharmaceutical applications as drug delivery systems, particularly in the treatment and diagnosis of cancer. Long-circulating liposomes are currently gaining recognition and clinical approval for their ability to improve drug delivery to target tissues. Liposomes have been acknowledged for their ability to selectively target diseased tissues through functionalization with targeting moieties. Furthermore, the appeal of liposomes lies in their biocompatibility and reduced drug clearance. Although liposomes are generally safe and intrinsically low in toxicity, efforts should be made to examine the environmental and toxicological impact of the long-term exposure of liposomes in humans and animals. Nevertheless, liposomes have proven to be promising drug delivery systems as evidenced by the widespread success of currently marketed liposomal products.

## Figures and Tables

**Table 1:** Liposomes functionalized with specific targeting ligands through various surface engineering techniques for the treatment of solid tumours.

Targeting ligand used	Surface modification method	Anticancer drug	Target tumour treated	Year
CA-IX antibody [51]	Coupling of DSPE-PEG-MAL micelles with DTT reduced antibodies was achieved via incubation for twenty-four hours at room temperature. DSPE-PEG-MAL-Ab micelles produced CA-IX directed liposomes by incubation at 60°C with preformed liposomes for two hours.	Docetaxel	Lung cancer	2014
Anti-HER2 antibody [52]	Terminal MAL group on liposomal surface conjugated to the thiol group	Doxorubicin	Breast cancer	2002
Anti-VEGFR2 [53]	Coincubation incorporated Fab'-Mal-PEG-DSPE into preformed liposomes for half an hour at 55°C succeeded by gel filtration purification	Doxorubicin	Colon cancer	2012
Anti-EGFR antibody [54]	The MAL group at the terminal end of DSPE-PEG-MAL preformed liposome chains was conjugated to thiolated antibody via incubation overnight with mild shaking at a temperature of 4°C	Small interfering RNA	Lung cancer	2015

**Table 2:** An illustration of the approved liposomal products currently on the market with their year of approval, intended clinical application, and preferred route of administration.

Drug name	Clinical application	Route of administration	Year of approval
Cytarabine and daunorubicin [26, 67, 68] (CPX-351)	Acute myeloid leukaemia	Intravenous injection	2017
Irinotecan [69, 70] (Onivyde®)	Pancreatic ductal adenocarcinoma (metastatic)	Intravenous injection	2015
Doxorubicin [25, 26, 71] (Lipo-dox)	Breast and ovarian cancer, Kaposi's sarcoma	Intravenous injection	2013
Vincristine [26, 72, 73] (Marqibo®)	Acute lymphoblastic leukaemia	Intravenous injection	2012
Exparel [74]	Bupivacaine	Intravenous injection	2011
Morphine Sulphate [26, 75] (DepoDur®)	Management of pain	Epidural	2004
Verteporfin [26, 76] (Visudyne®)	Sensitizer in photodynamic therapy for wet macular degeneration	Intravenous injection	2000
Doxorubicin [26, 77] (Myocet®)	Breast cancer	Intravenous injection	2000
Cytarabine [26, 78, 79] (DepoCyt®)	Lymphomatous meningitis	Intravenous injection	1999
Amphotericin B [26, 80, 81] (AmBisome®)	Fungal infections	Intravenous injection	1997 (USA) and 1990 (Europe)
Amphotericin B [26, 82] (Amphotec®)	Aspergillosis (Invasive)	Intravenous injection	1996
Daunorubicin [26, 83] (DaunoXome®)	Kaposi's sarcoma	Intravenous injection	1996
Amphotericin B [26, 84] (Abelcet®)	Aspergillosis	Intravenous injection	1995
Doxorubicin [26, 85–88] (Doxil®/Caelyx®)	Kaposi's sarcoma	Intravenous injection	1995

**Table 3:** An illustration of liposomal products in varying clinical trial phases yet to be approved with their intended clinical application and preferred administration route.

Drug name	Clinical application	Route of administration	Clinical trial phase	Year of approval
Doxorubicin [26, 89–91] (ThermoDox®) (thermosensitive)	Primary hepatocellular carcinoma	Intravenous injection	Phase III	2000
Cisplatin [26, 92, 93] (Lipoplatin^TM^)	Non-small cell lung cancer	Intravenous injection	Phase III	2007
BLP25 vaccine [26, 94, 95] (Stimuvax®)	Non-small cell lung cancer	Intravenous injection	Phase III	2006
Paclitaxel [26, 96, 97] (EndoTag®-1) (cationic)	Pancreatic cancer	Intravenous injection	Phase II	2005
L-Annamycin [74, 98]	Refractory acute AML or relapsed AML	Intravenous injection	Phase II	2005
Cisplatin [99] (SPI-77)	Head and neck cancer (*Z*)/lung cancer	Intravenous injection	Phase II	1998
Lurtotecan [100] (OSI-211)	Head, neck, and ovarian cancer	Intravenous injection	Phase II	2001
L-NDDP [101] (Aroplatin^TM^)	Colorectal cancer (metastatic)	Intravenous injection	Phase II	2002
Paclitaxel [102, 103] (LEP-ETU)	Solid tumour cancers	Intravenous infusion	Phase II	2004
Vinorelbine [104] (NanoVNB®)	Solid tumour cancers (advanced)	Intravenous injection	Phase I	2004
Mitoxantrone [74, 105] (LEM-ETU)	Solid tumour cancers (advanced)	Intravenous injection	Phase I	2001
Docetaxel [106] (ATI-1123)	Solid tumour cancers (advanced)	Intravenous injection	Phase I	2009
PKN3 targeting siRNA [107] (Atu027)	Solid tumour cancers (advanced)	Intravenous infusion	Phase I	2009
P53 (gene) [108] (SGT-53)	Solid tumour cancers	Intravenous infusion	Phase I	2008

## Data Availability

No data were used in the study.
